# SAPS 3 score as a predictive factor for postoperative referral to intensive care unit

**DOI:** 10.1186/s13613-016-0129-5

**Published:** 2016-04-30

**Authors:** João M. Silva, Helder Marcus Costa Rocha, Henrique Tadashi Katayama, Leandro Ferreira Dias, Mateus Barros de Paula, Leusi Magda Romano Andraus, Jose Maria Correa Silva, Luiz Marcelo Sá Malbouisson

**Affiliations:** 1 Hospital Servidor Publico Estadual-SP, Rua Pedro de Toledo, 1800/6º A–Vila Clementino, São Paulo, SP 04039-901 Brazil; 2 Anaesthesiology Department, Hospital das Clinicas SP-FMUSP, Av. Dr. Enéas de Carvalho Aguiar, 255 Cerqueira César, São Paulo, SP 05403-000 Brazil

**Keywords:** Surgical patients, Risk, Intensive care unit, Criteria

## Abstract

**Background:**

Patients undergoing intermediate-risk surgery are typically taken to the ward postoperatively. However, some may develop complications requiring intensive care later. We aimed to evaluate the characteristics of patients undergoing intermediate-risk surgery who required late postoperative admission to the intensive care unit (ICU) and determine the predictors for this.

**Methods:**

The study included patients undergoing intermediate-risk surgery with preoperative indication for ICU but who were taken to the ward postoperatively, because they appeared to be responding well. However, they required late ICU admission. ICU care and preoperative SAPS 3 score were evaluated. Palliative surgeries and patients readmitted to ICU were excluded.

**Results:**

The study included 100 patients, 27 % of whom had late postoperative admission to the ICU. The preoperative SAPS 3 score was higher (45.4 ± 7.8 vs. 35.9 ± 7.4, *P* < 0.001) in patients who required delayed admission to the ICU postoperatively. Furthermore, they had undergone longer surgery (4.2 ± 1.9 vs. 2.7 ± 1.5 h, *P* < 0.001), and a greater proportion were gastrointestinal surgeries (14.8 vs. 5.5 %, *P* = 0.03) and intraoperative transfusion (18.5 vs. 5.5 % *P* = 0.04). In multivariate analysis, preoperative SAPS 3 and surgery duration independently predicted postoperative ICU admission, respectively (OR 1.25; 95 % CI 1.1–1.4 and OR 3.33; 95 % CI 1.7–6.3).

**Conclusion:**

The identification of high-risk surgical patients is essential for proper treatment; time of surgery and preoperative SAPS 3 seem to provide a useful indication of risk and may help better to characterize patients undergoing intermediate-risk surgery that demand ICU care.

## Background

A substantial proportion of ICU patients are surgical patients. Nevertheless, high-risk surgical patients are not often identified as such and may experience a more difficult recovery postoperatively [1, 2]. A large observational study has indicated that surgical procedures classified as high risk have a mortality rate as high as 80 % [3]. Although less than 15 % of patients who underwent those procedures were admitted to an intensive care unit (ICU), the individual risk is often underestimated and high-risk patient factors may be overlooked.

As in the rest of the world, the scarcity of ICU beds in Brazil is one of the most important limiting factors for admission to ICU for an eligible patient [2, 4]. Patients with a real chance of recovery are thus prioritized [5, 6]. Surgical patients well illustrate this point, particularly those undergoing elective surgery [2]. The surgical outcome of that population is influenced not only by preoperative physiological status and surgical risk but also by adequate postoperative care [7]. Thus, it is paramount to know the predictors of the risks of increased morbidity and mortality for this group of patients [8].

Aiming for a better use of available resources, the Society of Critical Care Medicine has established criteria for the admission and discharge in ICUs [5, 6] to triage patients who may benefit most from intensive care. The proper use of such criteria is, however, not widespread, particularly in surgical patients.

Several other studies [8–11] have developed prognostic scores for critical patients and even for surgical ones [2, 7, 12, 13]. However, such scores have never been used preoperatively to decide who would or would not require an ICU bed. One of those indices is the SAPS 3 prognostic system [14]. This consists of 20 easily measured parameters [15], and its results, when utilized on high-risk surgical patients, are excellent [16]. Others, such as the ASA physical status index [17], are limited in predicting worse outcomes.

The performance of prognostic models encompasses two objective measures: calibration and discrimination. Calibration refers to how closely the estimated probabilities of mortality correlate with observed mortality over the range of probabilities. Discrimination refers to how well the model discriminates between individuals who will live and those who will die. From the individual patient’s point of view, a perfect discrimination would be preferable; however, for clinical trials or comparison of care between ICUs, better calibration is needed. Our intention with this study was to test the discriminatory power of preoperative SAPS 3 scores for ICU indication.

The study objective is thus to evaluate the characteristics and preoperative SAPS 3 scores of surgical patients undergoing intermediate-risk surgery, and who, owing to a decision by the surgical team, were referred to the ward postoperatively, but because of delayed postoperative complications, were admitted to ICU only later. The factors underlying such complications were also investigated.

## Methods

The study was approved by the Research Ethics Committee and exempted from the signed informed consent form requirement, because it was a case–control medical record review.

**Fig. 1:**
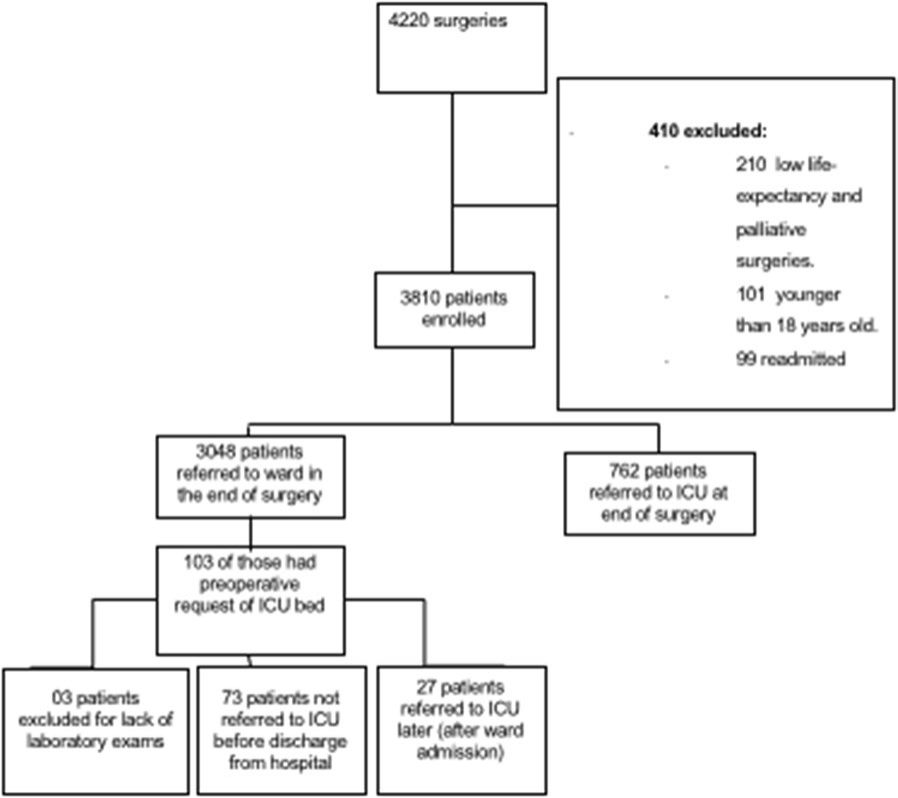
Flow chart of the number of patients screened

The patients included in the study were those undergoing intermediate-risk surgery, defined as those for whom an ICU bed was requested at preoperative assessment for postoperative care but who were not admitted to ICU postoperatively owing to clinical evaluation at the end of the operation. Patients under the age of 18 at the time of hospitalization, patients readmitted to ICU, and those who underwent palliative surgery were excluded from the study (see Fig. 1).

Clinical evaluation performed at the end of surgery included spontaneous breathing, a tidal volume of at least 6–8 ml/kg and respiratory a frequency of less than 25 breaths per minute, as well as peripheral oxygen saturation over 95 % and hemodynamic stability without vasopressor support. These patients needed to be alert and fully oriented, or, in case of preoperative cognitive impairment, the level of consciousness and orientation needed to be the same preoperatively. Other criteria included lack of bleeding, satisfactory control of pain, mobilization (as far as possible), spontaneous micturition, infection parameters within normal range, and non-irritated wound conditions. Patients were evaluated based on these criteria by surgeons and anaesthesiologists who were not involved neither in the study design nor in-group allocation.

Patients who had their medical records reviewed were divided into two groups, those who had an uneventful recovery in the surgical unit and those who, owing to late postoperative complications, had to be admitted to the ICU. The reviewed data allowed the research team to calculate the preoperative SAPS 3 score [14, 18] and the ASA physical status [17] of patients in both groups, as well as to define their demographics, the operative procedure they underwent, operative time, whether intraoperative blood transfusion was required, and hospital mortality. To calculate the SAPS 3 score, physiological data and laboratory analysis were performed on the day before surgery. Records were reviewed from hospitalization to medical discharge or hospital mortality.

At the time of the study, there was no official directive regarding the policy of triage and reservation of ICU beds. Patients were referred to the ICU at the surgical team’s discretion based on their experience.

### Statistical analysis

According to clinical experience and using the minimal clinically significant difference between groups for calculation, a sample of at least 93 participants would be required to produce a 10 % chance of an alternative hypothesis (late ICU admission) and a 2 % chance of a null hypothesis, accepting a type I error of 0.05 (one-sided) and power of 0.95.

Data were analysed, and the results are expressed as mean ± standard deviation, median (interquartile range), or percentage. For statistical analysis, variables, which followed a Gaussian distribution, were evaluated for significance by using the *t* test. Categorical variables were evaluated by the Chi-square test of contingency.

Binary logistic regression analysis was also performed, applying stepwise selection with backward elimination in order to identify independent risk factors and control confounding effects (variables mutually adjusted). Variables with significant probability (*P* value) of less than 0.05 in univariate analysis were considered candidates and removed in each step in the regression model if they presented a probability (*P* value) higher or equal to 0.10, by likelihood ratio test. Thereafter, selected variables for the regression model were tested to evaluate pairwise interaction possibilities, and those variables with interactions were corrected in the main regression model. If no statistically significant differences were found, variables were excluded. A bootstrap procedure based on 1000 bootstrap samples was applied in the main regression model to investigate the stability of coefficients and predictive ability of the variables included in model. The *P* values presented are from two-tailed tests, and values under 0.05 were considered statistically significant. Odds ratio and 95 % confidence interval (CI) were estimated by logistic regression. A ROC curve was determined for each variable. The cut-off points were estimated by the best sensitivity and specificity for ICU admission.

The Statistical Package for Social Sciences (SPSS-IBM Corp., Armonk, NY, USA) 21.0 was the software used for the statistical analyses.

## Results

From June 2013 to December 2013, a total of 4220 patients underwent surgery at the surgical centre of the hospital. Of these patients, 413 were excluded from the study. A total of 100 records were reviewed (see Fig. 1). The mean age of the patients was 66.4 years, with a standard deviation of 14.7 years. Their average SAPS 3 score was 38.5 ± 8.6 and 71 % were classified as ASA physical status 2. In total, 66 % were women. Elective surgery accounted for 84 % of cases, 30 % of patients had undergone gynaecological procedures, 28 % of surgeries were orthopaedic, and 16 % vascular. In the cases reviewed, neuraxial block was the most used type of anaesthesia, 3 % of patients died during hospitalization, and 27 % had a delayed referral to the ICU (see Table 1).

**Table 1:** Demographics of the patients included in the study

Variable	Value
Age	66.4 ± 14.7
SAPS 3 preoperatively	38.5 ± 8.6
ASA physical status P1 %	12.0
ASA physical status P2 %	71.0
ASA physical status P3 %	17.0
Female gender %	66
Elective surgery %	84.0
Emergency surgery %	16.0
Surgical speciality %	
Gynaecology	30.0
Orthopaedics	28.0
Vascular surgery	16.0
Urological surgery	13.0
Gastrointestinal surgery	8.0
Others	5.0
Postoperative haemoglobin (mg/dL)	12.9 ± 1.5
ICU admission %	27.0
PRBC transfusion %	9.0
Hospital mortality %	3.0
Anaesthesia %	
General anaesthesia	39.0
Neuraxial anaesthesia	49.0
General plus neuraxial anaesthesia	12.0

*ICU* intensive care unit, *PRBC* packed red blood cell transfusion; all values represent mean ± standard deviation, *Others* neurosurgery, head and neck surgery, thoracic surgery

When comparing both groups, SAPS 3 scores were higher among patients admitted to the ICU from the surgical unit (mean 45.4 ± 7.8) than among those who completed their recovery without ICU referral (mean 35.9 ± 7.4, *P* < 0.001). In the group of patients referred to critical care, 40.7 % were considered to be in ASA physical status 3, while only 8.2 % of the patients in the other group fitted that category (*P* = 0.001). In total, 18.5 % of patients in the delayed critical care group had received packed red blood cell transfusion intraoperatively, compared to only 5.5 % of patients who did not need intensive care (*P* = 0.004) (see Table 2).

**Table 2:** Comparison between patients who had uneventful recoveries in the surgical unit and patients who were referred to the ICU

Variables	ICU delayed (*n* = 27)	Non-ICU (*n* = 73)	*P*
Age (years)	65.7 ± 16.1	66.7 ± 14.3	0.75
Male gender (%)	33.3	34.2	0.93
Female % (%)	66.7	65.8	
SAPS 3	45.45 ± 7.8	35.94 ± 7.4	0.000
ASA (%)			0.002
P1	11.1	12.3	
P2	48.1	79.5	
P3	40.7	8.2	
Surgery (%)			
Elective	81.5	84.9	0.67
Emergence	18.5	15.1	0.29
Type of surgery (%)			
Gynaecology	11.1	37.0	0.02
Orthopaedic	22.2	30.1	0.59
Vascular	22.2	13.7	0.47
Urological	18.5	11.0	0.51
Gastrointestinal	14.8	5.5	0.26
Others	11.1	2.8	0.24
Anaesthesia (%)			0.11
General	55.6	32.9	
Neuroaxis	33.3	54.8	
General + neuroaxis	11.1	12.3	
Surgery time (h)	4.2 ± 1.8	2.7 ± 1.5	0.000
Transfusion requirements intraoperatively (%)	18.5	5.5	0.04
Value of haemoglobin immediately postoperatively(mg/dL)	12.5 ± 1.6	12.9 ± 1.5	0.20
Hospital mortality (%)	11.1	0.0	0.004
Length of hospital stay (days)	23.0 ± 22.2	25.8 ± 31.7	0.410

*ICU* intensive care unit, *Others* neurosurgery, head and neck surgery, thoracic surgery; all values represent mean ± standard deviation

In the multivariate analysis, the SAPS 3 score was determined as an independent factor for ICU referral (OR 1.25, 95 % CI 1.1–1.4, *P* = 0.001), as well as operative time (OR 3.33, 95 % CI 1.7–6.3, *P* < 0.001) (see Table 3).

**Table 3:** Logistic regression for delayed ICU

	*P*	OR	95 % CI		Bootstrap 95 % CI	
			Lower	Upper	Lower	Upper
SAPS 3 (per unit)	0.001	1.253	1.102	1.425	1.24	1.893
Surgery time (per hour)	0.000	3.327	1.750	6.325	2.235	8.618
ASA physical status (per unit)	0.991	0.991	0.207	4.749	−1.926	6.703
Transfusion requirements	0.593	1.737	0.230	13.145	−1.128	18.526
Gynaecological surgery	0.270	0.199	0.011	3.491	−19.662	4.161

The variables were adjusted in model by ASA, transfusion and surgery. Unless otherwise stated, the bootstrap results are based on 1000 bootstrap samples

**Fig. 2:**
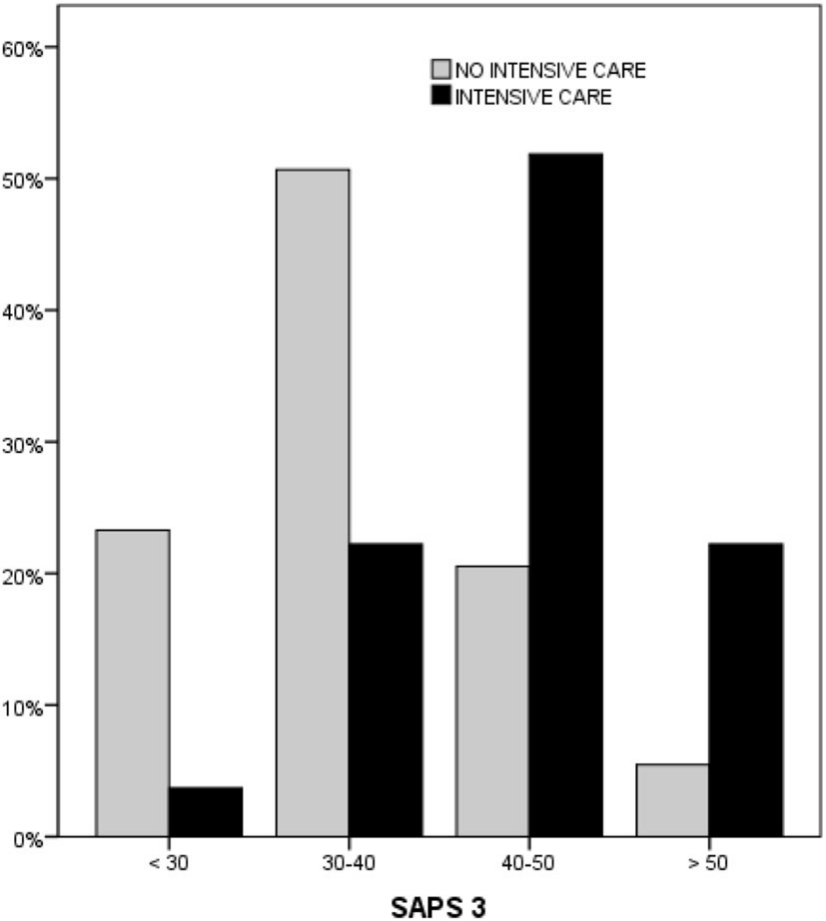
ROC curves of surgery time and SAPS 3 score for ICU referral

The ROC curve area was 0.87 (95 % CI 0.78–0.93) with a 44.2 cut-off point (sensitivity: 63 %; specificity: 87.7 %). For operative time, the ROC curve area was 0.78 (95 % CI 0.689–0.859), with a 3-h cut-off point (sensitivity: 74.1 %; specificity: 83.6 % (see Fig. 2).

**Fig. 3:**
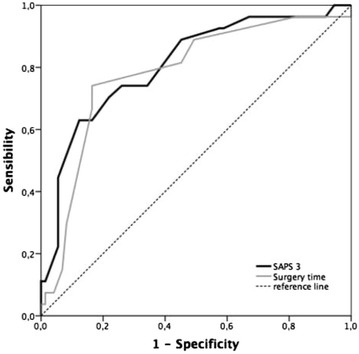
SAPS 3 score stratification and ICU referral rate

Patients with a preoperative SAPS 3 score exceeding 40 had a higher rate of ICU referral (see Fig. 3).

## Discussion

The identification of intermediary-risk surgical patients likely to require ICU care is difficult. To optimize postsurgical care and to prevent under and overuse of ICUs, criteria must be developed. We found that SAPS 3 scores and operative time were significantly higher in patients who eventually require critical care, showing that they could be used to predict early postoperative ICU necessity.

Predicting postoperative ICU need is complex in surgical patients. There are no self-evident criteria because many patient- and procedure-related factors determine ICU need. Because of the lack of objective criteria, many unnecessary admissions occur [11]. In this study, special attention was given to identifying which criteria could be used in practice to triage surgical patients and to determine which population found itself at higher surgical risk.

In multivariate analysis, predictive factors for delayed postoperative ICU admission were SAPS 3 score (OR 1.25 IC 95 % 1.1–1.4) and operative time (3.33 IC 95 % 1.7–6.3). In a recent study, Silva et al. [16] demonstrated the applicability of SAPS-3 in Brazilian hospitals, supporting its validation for surgical patients, consistent with other studies [13]. SAPS 3 is distinct from other prognostic scores because it is simple and does not require the use of sophisticated technological resources or complex data. Operative time is also easily obtained from the anaesthesia record.

Further studies have reached the same conclusion: postoperative risk is easily underestimated in clinical evaluation immediately postoperatively [3, 19]. In our study, 27 % of patients for whom an ICU bed had been requested but who were not taken to the ICU because they were evaluated as well enough to be cared for in the surgical unit, eventually had to be admitted to the ICU. Their postoperative complications might have been avoided if the request for an ICU bed had been met. This high incidence of late admission to the ICU illustrates the low predictive quality of the criteria used by the surgical team when deciding where the patient should be during the postoperative period, although the rate of refused ICU admissions for surgical patients is lower than that for non-surgical ones [20, 21].

For non-surgical patients, the age, severity of illness, and medical diagnosis are independent factors for ICU admission [22]. For surgical patients, it has been demonstrated that peritonitis, unplanned surgery, age, elevated serum lactate level, and a high central venous pressure on the day of ICU admission were independent predictors of death as a result of multiple organ failure [23]. Most of those factors are measured postoperatively. Identifying early predictors of postoperative complications might assist in the development and implementation of preventive measures.

The importance of preoperative planning is evident in the higher mortality rate of patients with late ICU admission. A late diagnosis and, therefore, a late referral to the ICU severely increase the chance of permanent damage to vital functions [23].

Clinical postoperative evaluation is too imprecise to identify surgical patients with a high risk of complications and may cause costly delays in adequate treatment. For 11 % of late admission patients in our study, this delay proved fatal.

Such complications result in longer hospitalizations, attended by problems such as sepsis, delirium, and organ failure [24]. Therefore, a clear set of objective criteria for ICU admission is important [6].

Findings indicate that subjective, individual analysis of a patient’s prognosis as well as isolated clinical parameters may lead to underestimation of the risk of complications and a delay in ICU referral [25].

Nowadays, there are few ICU admission guidelines [2, 4], and they are not widely utilized in clinical practice, particularly in the case of surgical patients.

The prediction of the SAPS 3 model is based exclusively on data evaluated within the first hour, which does not the case with other scores. Besides, the predictive power of the original SAPS 3 score is derived from information evaluated before ICU admission. Prognostic systems that include measurement up the first 24 h of ICU period are unavailable for use in the ICU screening. Furthermore, values above 24 h often capture the standard of care more than the actual clinical state of the patient; in this case, for example, the SOFA or APACHE scores may fall. This is the greatest advantage of SAPS 3, confirming its superiority over other prognostic scores in screening ICU patients. Other scoring systems, such as ASA-PS, do not include variables specific to the surgical procedure and are disadvantageous in their subjectivity.

The current study has some limitations. It is an observational study, which makes renders the potential for error high, even though enough statistical power was achieved. Another limitation is that it was conducted at a single surgical centre and that the sample size is small. Likewise, five variables were included in a logistic regression which evaluates only 27 events, but a bootstrap procedure was made based on 1000 bootstrap samples in order to investigate the stability of coefficients and predictive ability of the variables included in model, and a small amount of variation was found while the number of samples had increased considerably. Additionally, variables might have been overlooked and should constitute the subject of further analysis. For example, all patients requiring ICU care preoperatively could be included; in this way, patients who eventually went to ICU as predicted, those who did not go to ICU but were later admitted and those who never had to go to ICU, might be compared. Although the model applicable to all types of surgery, we have no data on its usefulness and predictability in other groups of patients. Further studies in different patient groups with different models are needed to determine the best predictive model.

## Conclusion

Preoperative SAPS 3 scores and surgery time are valuable tools for predicting the severity of illness and the risk of postoperative complications that might require intensive care. They were the most relevant factors analysed in this study.

More studies are required on this subject. The cost-effectiveness of precise indications of postoperative intensive care justifies further research.
