# Erratum to: SAPS 3 score as a predictive factor for postoperative referral to intensive care unit

**DOI:** 10.1186/s13613-016-0180-2

**Published:** 2016-08-16

**Authors:** João M. Silva, Helder Marcus Costa Rocha, Henrique Tadashi Katayama, Leandro Ferreira Dias, Mateus Barros de Paula, Leusi Magda Romano Andraus, Jose Maria Correa Silva, Luiz Marcelo Sá Malbouisson

**Affiliations:** 1 Hospital Servidor Publico Estadual-SP, Rua Pedro de Toledo, 1800/6º A–Vila Clementino, São Paulo, SP 04039-901 Brazil; 2 Anaesthesiology Department, Hospital das Clinicas SP-FMUSP, Av. Dr. Enéas de Carvalho Aguiar, 255 Cerqueira César, São Paulo, SP 05403-000 Brazil

## Erratum to: Ann Intensive Care (2016) 6:42 DOI 10.1186/s13613-016-0129-5

The original version of this article [1] unfortunately contained a mistake. The figures supplied for Figs. 2 and 3 were interchanged.

The corrected Figs. 2 and 3 and associated legends are supplied below.

**Fig. 2:**
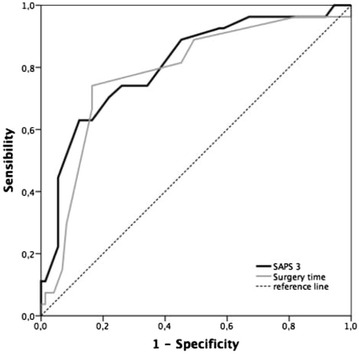
ROC curves of surgery time and SAPS 3 score for ICU referral

**Fig. 3:**
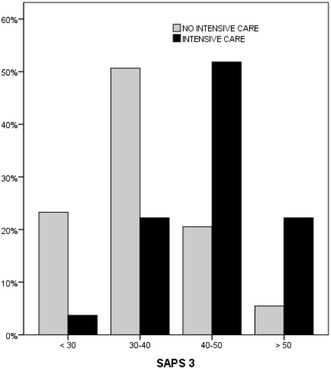
SAPS 3 score stratification and ICU referral rate
